# Association between relative fat mass and gallstones: a cross-sectional study based on NHANES 2017–2020

**DOI:** 10.3389/fnut.2025.1554659

**Published:** 2025-02-19

**Authors:** Chaofeng Gao, Yanan Li, Xuan Ren, Wei Han

**Affiliations:** ^1^Department of General Surgery, Lanzhou University Second Hospital, Lanzhou, China; ^2^Department of Anesthesiology, Lanzhou University Second Hospital, Lanzhou, China; ^3^Department of Endocrinology and Metabolism, Lanzhou University Second Hospital, Lanzhou, China

**Keywords:** relative fat mass, gallstones, cross-sectional study, NHANES, obesity

## Abstract

**Background:**

Gallstones are a common gastrointestinal disease worldwide, associated with significant public health burdens. Obesity and fat distribution are recognized as major risk factors for gallstone formation, yet traditional anthropometric indices such as BMI and WC have limitations in reflecting fat distribution and its metabolic consequences. Relative Fat Mass (RFM), a novel anthropometric index, may provide more accurate predictions of gallstone risk, but its association with gallstone formation remains underexplored.

**Methods:**

This study utilized data from NHANES 2017–2020, including 6,084 participants aged ≥20 years, to investigate the relationship between RFM and gallstone risk. Multivariable logistic regression and smooth curve fitting were used to assess this association. RFM’s predictive ability was compared with traditional indices using ROC and decision curve analysis (DCA). LASSO regression and AIC-based multivariable regression were employed to construct a gallstone risk prediction model.

**Results:**

Each one-unit increase in RFM was associated with a 11% higher risk of gallstones (OR: 1.11; 95% CI: 1.08–1.13). The smooth curve fitting revealed a linear relationship between RFM and gallstones. RFM demonstrated superior predictive ability (AUC = 0.705) compared to BMI, WC, WWI, and BRI. The predictive model, incorporating age, RFM, diabetes, waist circumference, and alcohol consumption, achieved good performance (AUC = 0.738) with sensitivity and specificity of 70 and 66%, respectively.

**Conclusion:**

RFM is strongly associated with gallstone risk and outperforms traditional anthropometric measures in risk prediction. The study presents a model that serves as a useful instrument for recognizing populations at elevated risk and facilitating focused interventions, especially among those with a prevalent occurrence of obesity and metabolic disturbances. These findings support the potential of RFM as an effective measure in clinical and public health settings for reducing the burden of gallstone-related diseases.

## 1 Introduction

Gallstones are a digestive system disease characterized by the formation of calculi in the gallbladder or bile ducts, primarily due to abnormally elevated levels of cholesterol or bilirubin in the bile (1). Globally, gallstones constitute one of the most common gastrointestinal disorders, affecting roughly 10 to 15% of the global populace, and they impose a significant burden on healthcare systems and societal costs (2, 3). In addition, the incidence of gallstones exhibits notable geographic variations, with distinct epidemiological patterns across different countries and regions (4). In the United States alone, an estimated 700,000 to 1 million cholecystectomy procedures are performed annually (5). Therefore, the prevention and early detection of gallstones represent a critical public health challenge. Multiple factors contribute to the development of gallstones, including age, sex, ethnicity, and conduct related to daily living such as dietary habits and physical exertion (6). In particular, the prevalence of gallstones is significantly higher in middle-aged women compared to young men, which may be related to changes in their estrogen levels (4). These changes in estrogen levels can reduce the contractile function of the gallbladder, thereby increasing the risk. Among these, obesity and metabolic abnormalities have increasingly been recognized as significant risk factors for gallstone formation (7), reflecting the high prevalence of metabolic diseases in modern society and their growing public health impact.

General anthropometric measurement indicators, such as body mass index (BMI) and waist circumference (WC), have long been utilized for a substantial period to assess obesity and the associated health risks. However, BMI cannot account for body fat distribution or overall body fat content, limiting its application in diagnosing obesity-related conditions (8). Although WC has been shown to be associated with visceral fat and abdominal obesity, it has limitations in accurately reflecting total body fat content and distribution (9). Emerging indices, such as relative fat mass (RFM), weight-to-waist index (WWI), and body roundness index (BRI), have attracted increasing attention due to their potential to provide more precise assessments of body composition (10–12). In particular, RFM, as a reliable estimate of body fat percentage that has been determined, demonstrating strong associations with various metabolic and cardiovascular ailments (13–15). Nevertheless, the connection between RFM and gallstones remains underexplored.

This study aims to examine the correlation between RFM and gallstone risk utilizing data from the 2017–2020 National Health and Nutrition Examination Survey (NHANES). Leveraging the nationally representative NHANES dataset, we seek to evaluate the forecasting ability of RFM compared to traditional indicators. Additionally, this study aims to develop a risk prediction model incorporating RFM and other clinical variables to provide practical tools for identifying high-risk populations and formulating effective prevention strategies.

## 2 Methods

### 2.1 Study population

This study employed a cross-sectional design to analyze the 2017–2020 NHANES dataset. NHANES utilizes a multi-stage stratified sampling strategy to systematically gather information regarding the nutritional and health status of U.S. community residents, ensuring national representativeness.

**Figure 1:**
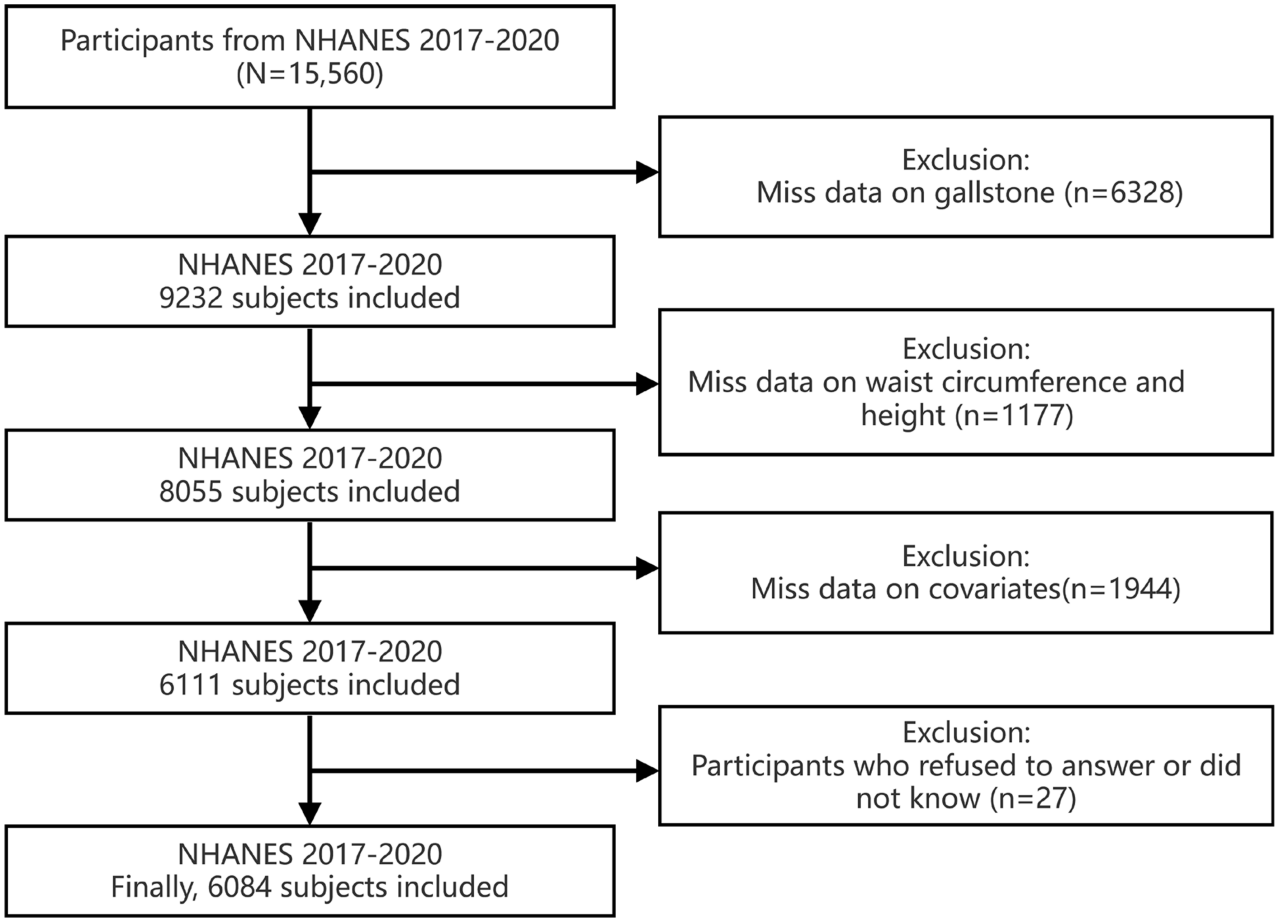
Flowchart of study cohort selection process.

Since gallstone-related questionnaires were only available during the 2017–2020 NHANES cycle, the data utilized in this research were confined to that specific timeframe. The following were the criteria for excluding participants: (1) individuals with missing information on gallstones; (2) participants with missing waist circumference or height data; (3) individuals with missing covariate data; and (4) participants who refused to answer or responded with “do not know.” Additionally, as the covariate for education level was collected only from participants aged 20 years and above, this study included only People who are 20 years of age or above. Ultimately, this study included 6,084 individuals, representing over 185.31 million people in the United States. The detailed screening steps are shown in Figure 1.

The NHANES study protocol was approved by the NCHS Research Ethics Review Board, and all participants provided written informed consent. Further data analysis does not require additional ethical approval or participant consent.

### 2.2 Assessment of RFM, WWI, BRI, and gallstone

RFM is used to assess body fat content, and its calculation formula is: RFM = 64 − (20 × Height/WC) + (12 × Gender), where females are assigned a value of 1 and males a value of 0 (10). WWI and BRI were also used as anthropometric indicators in the assessment of gallstone risk (16, 17). The calculation method for WWI is: WWI = weight (kg) /waist circumference (m^3^) (18); for BRI, it is: BRI = 364.2–365.5 × √(1 − waist circumference^2^/height^2^) (19). All relevant measurement data, including weight, height, and waist circumference, were gathered via the Mobile Examination Center (MEC). The presence of gallstones was assessed using a questionnaire, with the specific question: “Has a doctor or other health professional ever told you that you have gallstones?” Participants who answered “yes” were classified as having gallstones.

### 2.3 Covariates

Based on previous studies (14, 20), this study selected several confounding factors as covariates, incorporating age, race, educational attainment, BMI, poverty-income ratio (PIR), total cholesterol (TC), the status of tobacco use, physical activity, alcohol intake, diabetic status, and hypertension. The specific definitions of the covariates are shown in Supplementary Table S1.

### 2.4 Statistical analysis

All statistical analyses were performed using R (version 4.3.3) and Empower software (version 4.2). To ensure the data were nationally representative, NHANES-provided sampling weights were applied. The means, accompanied by standard deviations (SD), were utilized to represent continuous variables, whereas categorical variables were depicted through unweighted frequencies and percentages with weights applied. Differences between groups were assessed using t-tests for continuous variables and chi-squared tests for categorical variables. To investigate the relationship between RFM and gallstones, three multivariable logistic regression models were constructed: Model 1 (unadjusted), Model 2 (adjusted for age, and race), and Model 3 (additional adjustments were made to account for educational attainment., BMI, smoking status, physical activity, alcohol consumption, hypertension, PIR, total cholesterol, and diabetes). The findings were presented in the form of odds ratios (OR), accompanied by 95% confidence intervals (CI) for statistical significance. Further, smooth curve fitting was used to explore whether there is a nonlinear relationship between RFM and gallstone risk. Detailed subgroup evaluations were conducted to assess the robustness of associations across different demographic and clinical subpopulations, such as age, ethnic group, education level, BMI, smoking status, diabetes, alcohol consumption, physical activity, and hypertension. Interaction terms were included to evaluate potential effect modifications, with statistical significance for interactions set at P for interaction <0.05. The objective is to evaluate the predictive prowess of RFM and other anthropometric indices (e.g., BMI, WC, WWI, BRI), receiver operating characteristic (ROC) curve analysis and decision curve analysis (DCA) were performed. Discriminative ability was assessed by calculating the area under the curve (AUC), while DCA was used to evaluate net benefits across different risk thresholds. Finally, to ascertain clinical variables that are notably correlated with the risk of gallstones, LASSO regression is employed. To evaluate the clinical variables pinpointed through LASSO regression, we proceed with both univariate and multivariate logistic regression analyses, utilizing the Akaike Information Criterion (AIC) for the purpose of model selection. A gallstone risk prediction model incorporating five variables—age, RFM, diabetes, waist circumference, and alcohol consumption—was constructed, and its performance was evaluated using ROC and DCA. We established statistical significance by setting a threshold for the two-tailed *p*-value <0.05.

## 3 Results

### 3.1 Baseline characteristics

This study ultimately included 6,084 participants with an average age of 48.00 ± 17.02 years, and an equal gender distribution (50% male and 50% female). Among the participants, 67% were Non-Hispanic White, with an average RFM value of 34.97 ± 8.63. Based on whether gallstones were present, the participants were categorized into two distinct groups, with 11% diagnosed with gallstones. We found significant differences in age, gender, educational level, PIR (Poverty Income Ratio), BMI, physical activity, smoking status, diabetes, drinking situation, and hypertension (*p* < 0.05) between the two groups of participants. Individuals affected by gallstones tended to be predominantly female, of advanced age, exhibit a higher BMI, possess a lower level of education, engage in lesser amounts of physical activity, have a reduced income, a history of smoking, a tendency to abstain from alcohol, and more frequently had diabetes or hypertension (Table 1).

**Table 1:** Study population characteristics.

Characteristic		Gallstone		
	Overall, *N* = 6,084 (100%)^a^	No, *N* = 5,438 (89%)^a^	Yes, *N* = 646 (11%)^a^	*p*-value^b^
Age (years)	48.00 ± 17.02	46.00 ± 16.93	59.00 ± 15.30	**<0.001**
Gender				**<0.001**
Male	3,091 (50%)	2,903 (52%)	188 (26%)	
Female	2,993 (50%)	2,535 (48%)	458 (74%)	
Race				0.072
Mexican American	694 (7.8%)	614 (7.9%)	80 (6.9%)	
Other Hispanic	583 (6.7%)	511 (6.7%)	72 (6.8%)	
Non-Hispanic White	2,343 (67%)	2046 (66%)	297 (72%)	
Non-Hispanic Black	1,594 (10%)	1,467 (11%)	127 (6.7%)	
Other race	870 (8.4%)	800 (8.5%)	70 (7.7%)	
Education level				**0.032**
≤ High school	2,433 (36%)	2,159 (35%)	274 (42%)	
> High school	3,651 (64%)	3,279 (65%)	372 (58%)	
BMI group (kg/m^2^)				**<0.001**
< 25	1,503 (26%)	1,433 (28%)	70 (12%)	
25–30	1895 (31%)	1718 (31%)	177 (31%)	
≥ 30	2,686 (43%)	2,287 (41%)	399 (58%)	
Physical activity				**0.034**
Yes	2,994 (56%)	2,731 (57%)	263 (49%)	
No	3,090 (44%)	2,707 (43%)	383 (51%)	
Smoking status				**<0.001**
Never	3,310 (55%)	2,996 (56%)	314 (46%)	
Former	1,592 (28%)	1,370 (27%)	222 (37%)	
Current	1,180 (17%)	1,071 (18%)	109 (17%)	
Diabetes				**<0.001**
Yes	1,190 (14%)	983 (13%)	207 (24%)	
No	4,894 (86%)	4,455 (87%)	439 (76%)	
PIR	3.39 ± 1.62	3.42 ± 1.63	2.92 ± 1.54	**0.003**
Alcohol				**<0.001**
Yes	3,147 (57%)	2,895 (59%)	252 (42%)	
No	2,937 (43%)	2,543 (41%)	394 (58%)	
Hypertension				**<0.001**
No	3,751 (68%)	3,450 (70%)	301 (51%)	
Yes	2,333 (32%)	1988 (30%)	345 (49%)	
TC (mg/dL)	4.76 ± 1.05	4.76 ± 1.05	4.81 ± 1.08	0.5
RFM	34.97 ± 8.63	34.21 ± 8.45	43.25 ± 7.70	**<0.001**

a Median ± SD for continuous; *n* (unweighted) (%) for categorical.

b *T-test adapted to complex survey samples; chi-squared test with Rao & Scott’s second-order correction.Bold values indicate statistically significant results at p < 0.05.*

### 3.2 The relationship between RFM and gallstones

To ensure the robustness of the model, we assessed the multicollinearity among the variables. The calculated Generalized Variance Inflation Factor (GVIF) values (see Supplementary Table S2) were all below 5, indicating no significant multicollinearity among the independent variables. Based on this, we proceeded to evaluate the relationship between RFM and gallstones using three multivariable regression models, with the results presented in Table 2. The findings suggested a correlation that is favorable between RFM and the presence of gallstones. In Model 1, without adjusting for any covariates, the relationship between RFM and gallstones was significant, with each one-unit increase in RFM associated with a 10% higher risk of gallstones (OR: 1.10, 95% CI: 1.09–1.12). In Model 2, following adjustments for age and race, the relationship between RFM and gallstones remained significant, with an OR of 1.10 (95% CI: 1.08–1.12). In Model 3, subsequent adjustments were made for factors including education level, BMI, smoking habits, physical activity, alcohol consumption, hypertensive status, PIR, TC, and diabetes, the association between RFM and gallstones remained significant, with an OR of 1.11 (95% CI: 1.08–1.13). An increment of one unit in the RFM score correlated with a 11% elevated probability of developing gallstones. To further clarify the complex relationship between RFM and gallstones, we divided RFM into quartiles for additional analysis. The results showed that individuals in the higher RFM quartiles, particularly in Q4, exhibited a significantly elevated risk of gallstones, with an OR of 5.99 (95% CI: 3.39–10.6) in Model 2 and 5.67 (95% CI: 2.47–13.0) in Model 3. The trend analysis identified a significant linear association between RFM quartiles and gallstone risk (Model 3: p for trend <0.001).

**Table 2:** The association between RFM and gallstones.

	Model 1 OR (95% CI)	Model 2 OR (95% CI)	Model 3 OR (95% CI)
RFM	1.10 (1.09, 1.12)	1.10 (1.08, 1.12)	1.11 (1.08, 1.13)
RFM			
Q1 (10.09,29.54)	Reference	Reference	Reference
Q2 (29.54,34.97)	1.64 (0.83, 3.22)	1.41 (0.71, 2.79)	1.39 (0.58, 3.32)
Q3 (34.97,42.95)	3.07 (1.84, 5.14)	2.67 (1.59, 4.49)	3.00 (1.55, 5.81)
Q4 (42.95,58.24)	7.27 (4.14, 12.7)	5.99 (3.39, 10.6)	5.67 (2.47, 13.0)
*P* for trend	**<0.001**	**<0.001**	**<0.001**

Model 1: no covariates were adjusted. Model 2: age and race were adjusted. Model 3: age, race, education level, BMI, smoking status, physical activity, alcohol, hypertension, PIR, TC and diabetes were adjusted.Bold values indicate statistically significant results at *p* < 0.05.

### 3.3 Smoothed curve fitting analysis

**Figure 2:**
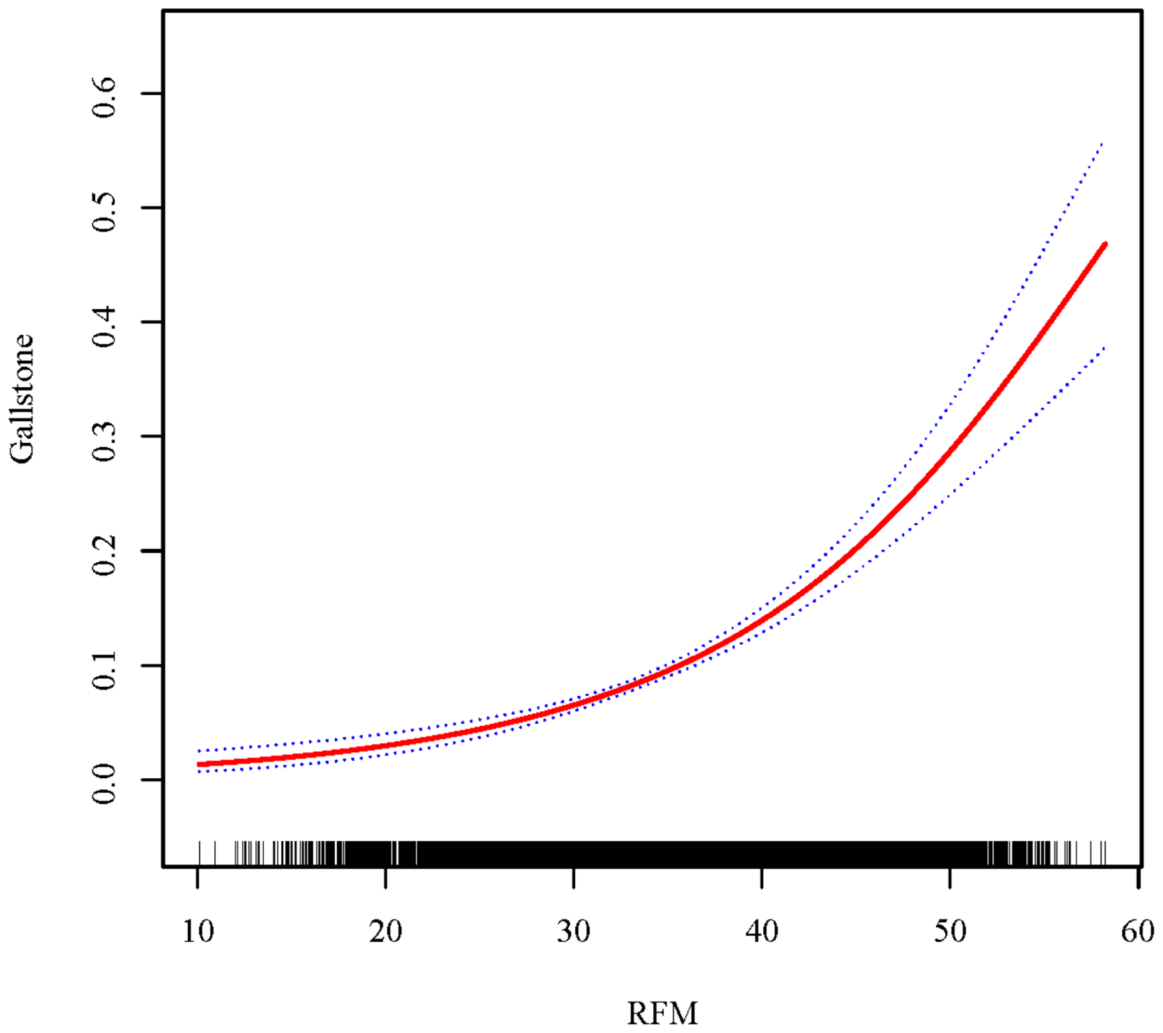
The association between RFM and gallstone.

To further explore and visualize whether a nonlinear relationship exists between RFM and gallstones, a smoothing curve was fitted after adjusting for all covariates (Figure 2). The results showed a positive linear trend between RFM and the likelihood of gallstone occurrence (log-likelihood ratio = 0.104). As RFM increased, the risk of gallstones gradually rose.

### 3.4 Subgroup analysis and interaction testing

**Figure 3:**
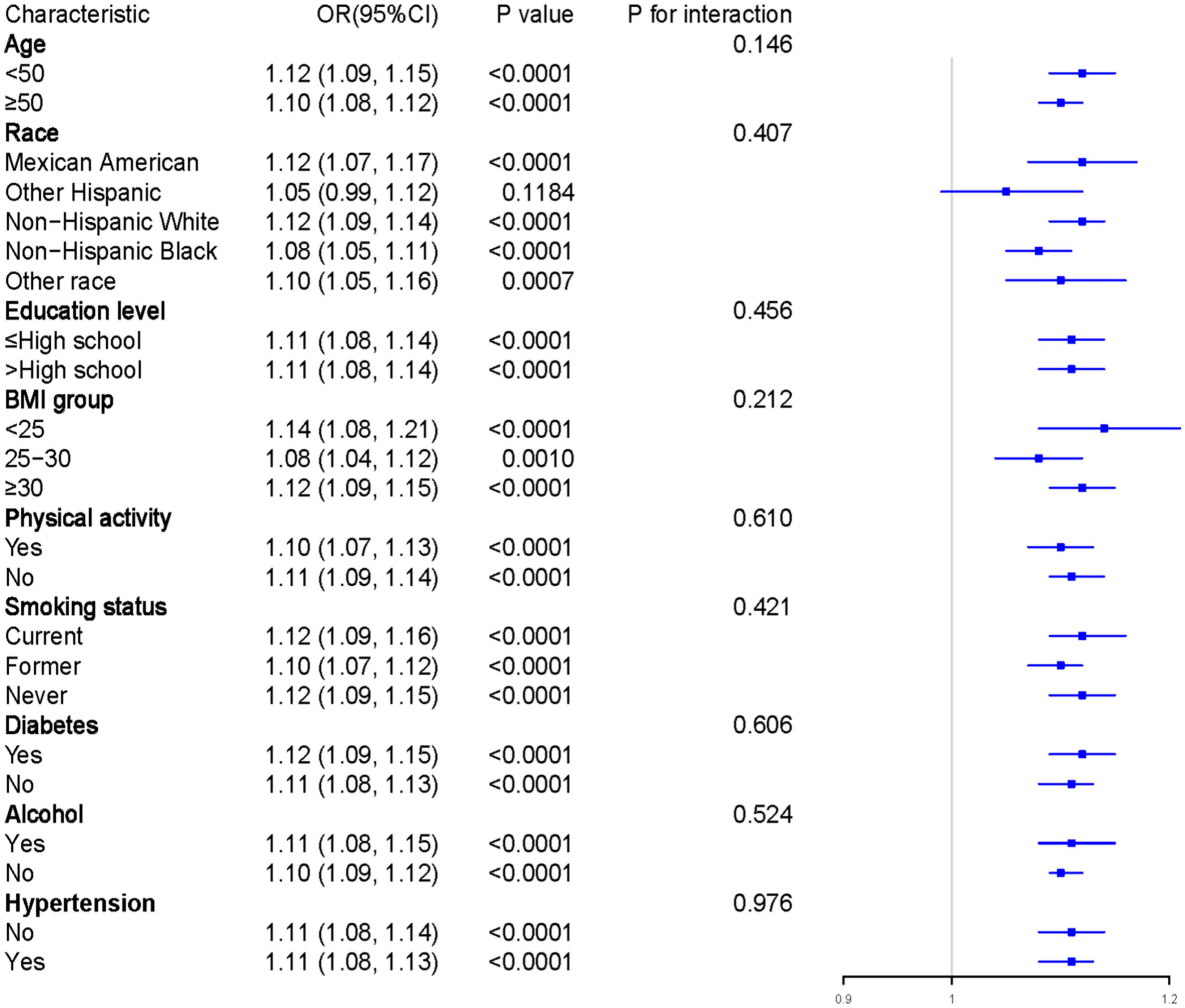
Results of subgroup analysis. Adjustments have been made for all covariates in the investigation. OR, odds ratio; CI, Confidence interval.

To assess the robustness and heterogeneity of the relationship between RFM and gallstones, we conducted a subgroup analysis on variables including age, race, education level, BMI, physical activity, smoking status, diabetes, alcohol consumption, and hypertension, with relevant confounding factors adjusted accordingly. As shown in Figure 3, except for certain racial subgroups where the association between RFM and gallstones did not exhibit a statistically significant positive correlation, significant positive associations were observed across other subgroups. However, interaction analysis results indicated that no statistically meaningful variations among the subgroups (P for interaction >0.05). Overall, the results demonstrate that the association between RFM and gallstones is robust across different subgroups.

### 3.5 ROC curve analysis

**Figure 4:**
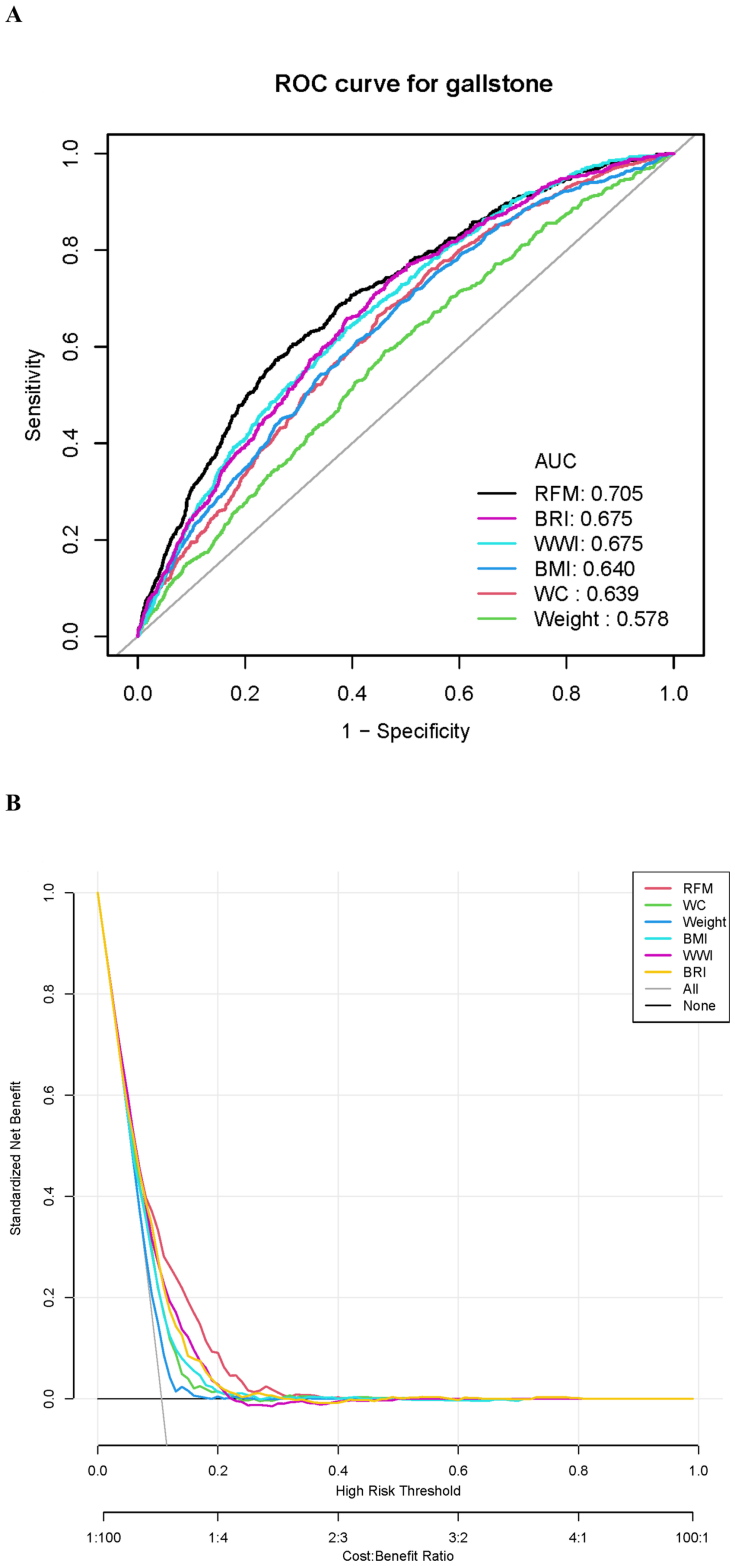
Evaluation of the predictive performance of RFM and other indices for gallstones. **(A)** ROC curve. **(B)** Decision curve analysis (DCA).

To assess the effectiveness of RFM and other indices in predicting the risk of gallstones, we conducted ROC curve analysis and DCA to comprehensively compare the predictive performance and clinical applicability of these indices. As illustrated in Figure 4A and Table 3, the AUC of RFM was 0.705, which was the best among all evaluated indices, significantly outperforming BRI (AUC = 0.675), WWI (AUC = 0.675), BMI (AUC = 0.640), WC (AUC = 0.639), and weight (AUC = 0.578). This result indicates that RFM has a higher discriminative ability in predicting the risk of gallstones. Further analysis through DCA (Figure 4B) assessed the net benefit of each index across different high-risk thresholds. The results showed that RFM had a significantly higher net benefit than other indices in most high-risk threshold ranges, particularly in the low to moderate risk threshold range (0.05–0.3), where its clinical benefit was the greatest. This suggests that, compared to traditional indices, RFM has greater practical value in screening and early prevention for high-risk populations of gallstones. Additionally, we assessed the model’s calibration using calibration curves (Supplementary Figure S1). These curves further support RFM’s performance in predicting gallstone risk, showing good alignment between predicted and actual probabilities. Especially compared to other indices, RFM demonstrated the best calibration and accuracy.

**Table 3:** Predictive performance of independent factors.

Variable	AUROC	95% CI	Sensitivity	Specificity
RFM	0.705	0.684–0.726	0.605	0.711
WC	0.639	0.618–0.660	0.664	0.552
Weight	0.578	0.555–0.601	0.591	0.538
BMI	0.64	0.618–0.662	0.612	0.591
WWI	0.675	0.654–0.695	0.639	0.611
BRI	0.676	0.655–0.696	0.738	0.531

RFM: relative fat mass; WC: waist circumference; BMI: body mass index; WWI: weight-to-waist index; BRI: body roundness index.

### 3.6 LASSO regression for feature screening

**Figure 5:**
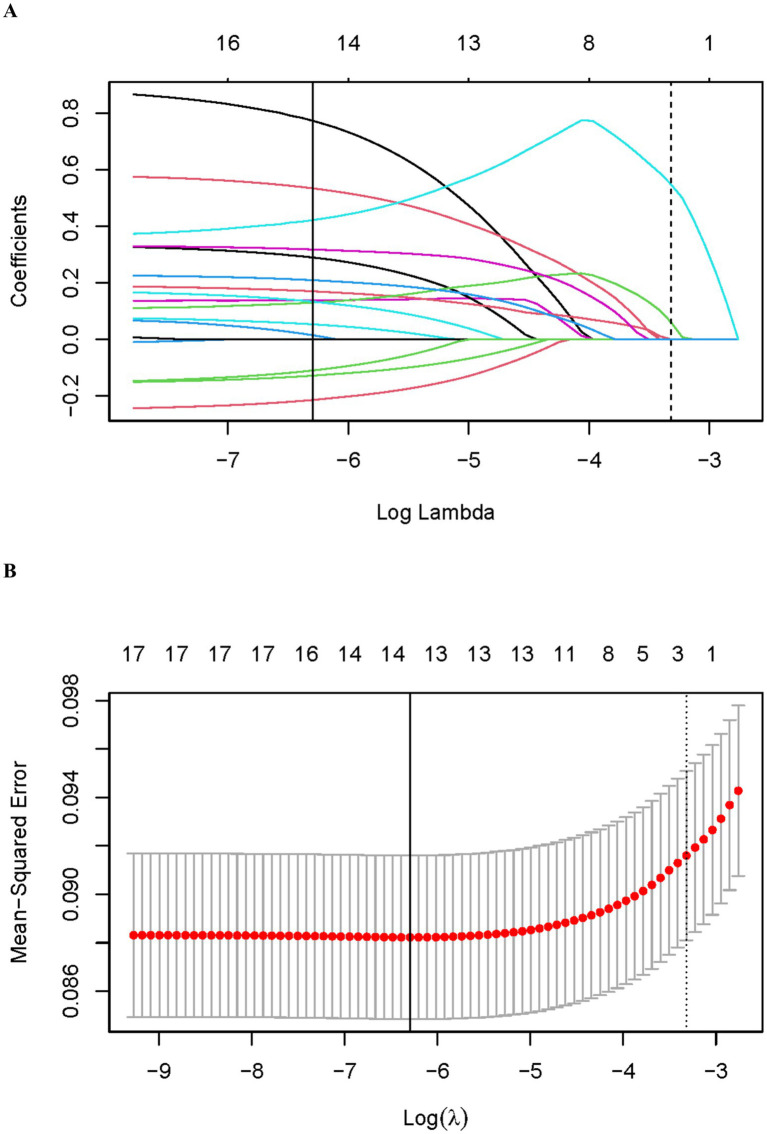
LASSO regression analysis for variable selection. **(A)** Coefficient profiles of variables. **(B)** Ten-fold cross-validation for LASSO regression.

To identify the best variables for predicting gallstone risk, we conducted LASSO regression analysis on the initially selected clinical variables (Figures 5A,B). By incorporating a penalty term, LASSO regression reduced variable redundancy and optimized the model’s stability and predictive performance. With the selection of the optimal penalty parameter (log *λ* = −5.696), 14 significant variables were identified, including age, gender, education level, BMI, waist circumference, weight, WWI, BRI, smoking status, diabetes, alcohol consumption, total cholesterol, hypertension, and RFM.

### 3.7 Univariate and multivariate analysis

The variables selected via LASSO regression underwent subsequent analysis employing univariate and AIC-based multivariate regression analyses (Table 4). The univariate regression analysis showed that age, gender, weight, WC, BMI, BRI, WWI, RFM, alcohol consumption, education level, diabetes, and hypertension were significantly associated with the risk of gallstones (*p* < 0.05). In the multivariate regression model, the final independent risk factors identified were: age (OR = 1.02, 95% CI = 1.02–1.03, *p* < 0.001), RFM (OR = 1.09, 95% CI = 1.07–1.10, *p* < 0.001), and diabetes (OR = 1.35, 95% CI = 1.10–1.66, *p* = 0.004). In the multivariate analysis, statistical significance was not achieved for waist circumference and alcohol intake (*p* > 0.05).

**Table 4:** Univariate and multivariate analysis.

Variable	Univariate analysis			Multivariate analysis		
	OR	95%CI	*p* value	OR	95%CI	*P* value
Age	1.03	1.02–1.03	**0.000**	1.02	1.02–1.03	**0.000**
Gender	2.79	2.32–3.35	**0.000**			
Weight	1.01	1.01–1.02	**0.000**			
WC	1.03	1.02–1.03	**0.000**	1.00	1.00–1.01	0.154
BMI	1.83	1.63–2.07	**0.000**			
BRI	1.24	1.2–1.27	**0.000**			
WWI	2.13	1.92–2.37	**0.000**			
RFM	1.09	1.08–1.11	**0.000**	1.09	1.07–1.10	**0.000**
Alcohol	0.57	0.48–0.68	**0.000**	0.84	0.70–1.01	0.058
Diabetes	2.13	1.77–2.56	**0.000**	1.35	1.10–1.66	**0.004**
Education	0.9	0.76–1.07	**0.000**			
Hypertension	1.97	1.67–2.34	**0.049**			
TC	0.97	0.9–1.05	0.484			
Smoke	1.07	0.96–1.19	0.212			

Bold values indicate statistically significant results at *p* < 0.05.

### 3.8 Construction and evaluation of a gallstone risk prediction model

**Figure 6:**
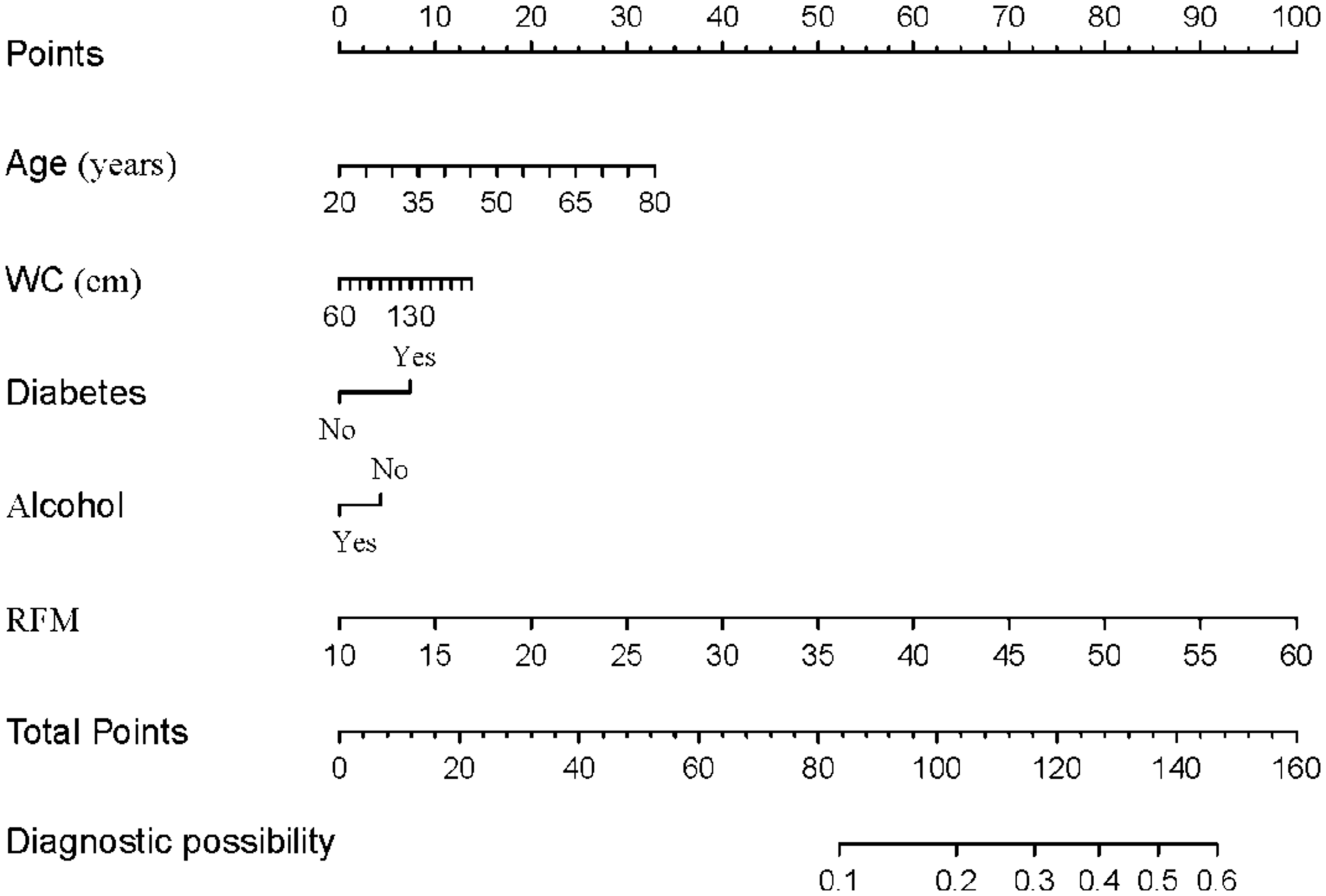
Nomogram for predicting the risk of gallstones.

**Figure 7:**
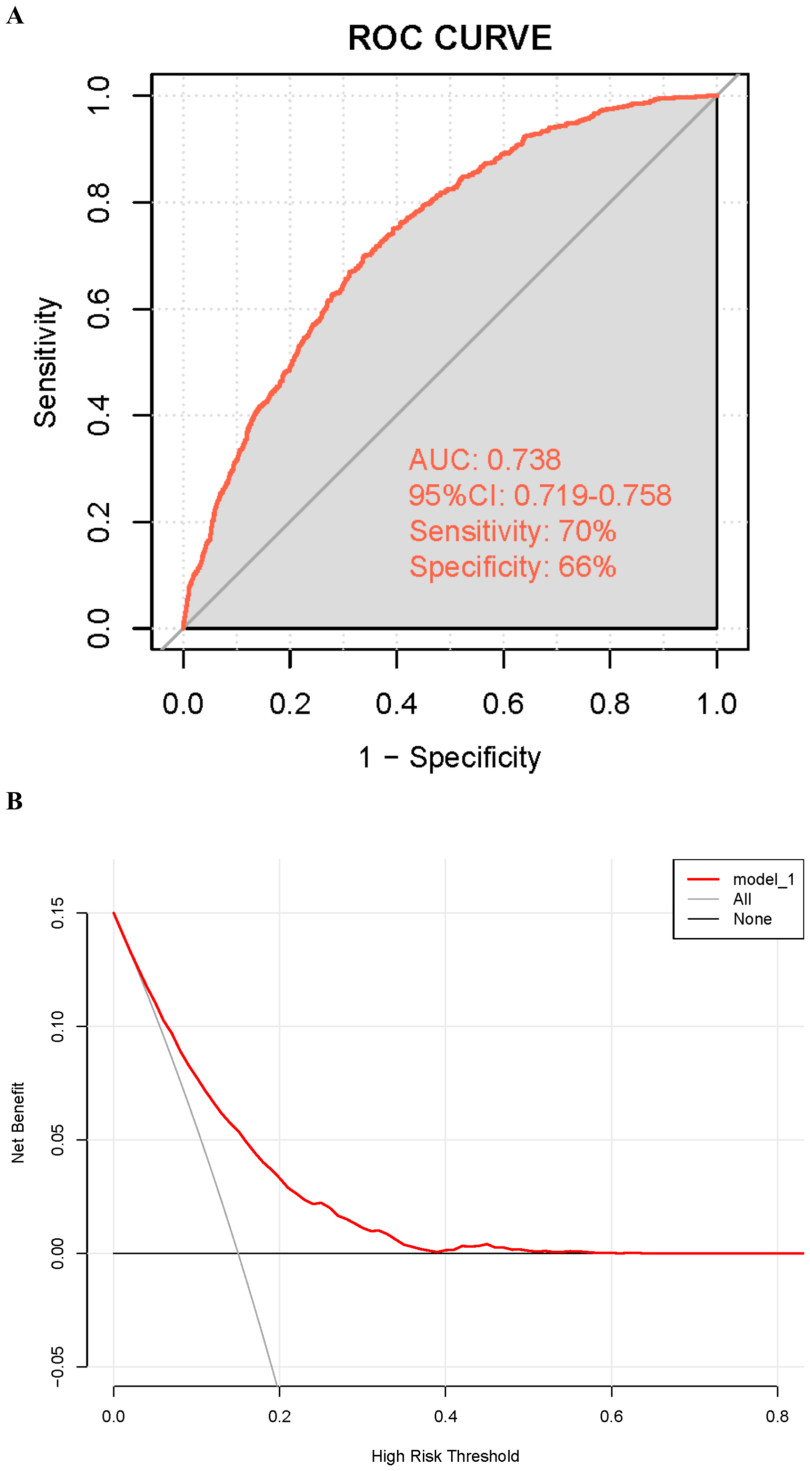
Evaluation of the predictive performance of the gallstone prediction model. **(A)** Receiver operating characteristic (ROC) curve. **(B)** Decision curve analysis (DCA).

Through univariate and multivariate logistic regression analyses, we initially identified variables significantly associated with the risk of gallstones. Based on multivariate analysis, we further optimized the model’s performance and reduced redundant variables by applying the AIC criterion. Ultimately, five variables—age, WC, diabetes, alcohol consumption, and RFM—were included to construct the predictive model (Figure 6). The detailed stepwise regression analysis process and variable coefficients are provided in Supplementary Table S3. The nomogram clearly illustrates the contribution of each variable to gallstone risk, providing a quantitative basis for individualized risk assessment. The performance of the model was evaluated using the ROC curve and decision curve analysis (Figures 7A,B). ROC analysis revealed an AUC value of 0.738 (95% CI: 0.719–0.758) for the model, indicating good discriminative ability. The model exhibited a sensitivity of 70% and a specificity of 66%, respectively, as indicated. Additionally, the decision curve analysis demonstrated that within the high-risk threshold range of 0.1–0.4, the model achieved a notably superior net benefit in comparison to the “treat-all” or “treat-none” approaches. Furthermore, calibration curves (Supplementary Figure S2) further confirmed the model’s good calibration, demonstrating excellent agreement between predicted and observed probabilities.

## 4 Discussion

This research systematically analyzed the association between RFM and the risk of gallstones based on NHANES 2017–2020 data and developed a gallstone risk prediction model. In the model adjusted for multiple variables, an increment of one unit in RFM was found to correlate with an elevated risk of gallstones by 11%. Smooth curve fitting showed a linear association between RFM and gallstone risk, with the likelihood of developing gallstones increasing as RFM rises. The stability of the positive correlation between RFM and gallstones was confirmed through subgroup analyses and this result remains consistent across different subgroups.

Additionally, the predictive capability of RFM was evaluated using ROC and DCA, showing an AUC of 0.705, which was significantly better than traditional indices such as BMI, WC, WWI, and BRI. RFM also demonstrated greater clinical net benefit in the low-to-moderate risk threshold range (0.05–0.3). Based on these findings, the study employed LASSO regression to select variables, combined with univariate and multivariate regression analyses, and employed the AIC criterion to ultimately identify five variables—age, RFM, diabetes, waist circumference, and alcohol consumption—for constructing the gallstone risk prediction model. The AUC attained by the model was 0.738, exhibiting a sensitivity of 70% and a specificity of 66%, indicating good predictive performance. These results not only confirm the significance of RFM as an independent risk factor for gallstones but also highlight the potential clinical value of integrating multivariable prediction models in the screening and prevention of gallstones in high-risk populations.

This research represents the initial investigation to establish a favorable correlation between RFM and gallstone risk, highlighting the potential role of obesity and fat distribution in the pathogenesis of gallstones. As a reliable indicator for evaluating whole-body fat percentage, RFM demonstrates a stronger ability to reflect the metabolic consequences of excessive fat accumulation compared to traditional measures such as BMI or WC (21). The linear relationship observed in this study suggests that higher levels of body fat may exacerbate the formation of gallstones by worsening metabolic dysfunction. It is well known that visceral fat accumulation is a critical marker of obesity-related metabolic dysfunction and it occupies a pivotal position in the underlying mechanisms leading to the formation of gallstones. Studies have shown that fat accumulation disrupts hepatic lipid metabolism, leading to an increase in cholesterol levels in bile (22). When cholesterol concentration in bile becomes excessively high and exceeds the solubility limits of bile acids and phospholipids, cholesterol crystals form, which in turn lead to the development of gallstones (23). Furthermore, the presence of visceral adipose tissue generates inflammatory mediators, including TNF-*α* and IL-6, which not only aggravate metabolic dysfunction but also impair gallbladder function. This results in bile flow obstruction, thereby promoting gallstone formation (24). Obesity-related metabolic disorders, such as insulin resistance, also play a significant role in gallstone formation (25). For example, Gong et al. exhibited that the TyG index, which serves as a marker for insulin resistance, is significantly associated with gallstone risk, with this relationship being more pronounced in obese and female individuals (26). Moreover, Wang et al. found that METS-IR, a more comprehensive and integrative indicator of insulin resistance compared to TyG, is also significantly associated with gallstone risk and is correlated with earlier occurrence of the first cholecystectomy (27). Insulin resistance increases gallstone risk through multiple mechanisms: not only does it elevate cholesterol levels in bile, but it also reduces the secretion of bile acids and phospholipids, both of which are crucial for preventing cholesterol crystallization in the gallbladder (28, 29). Additionally, recent studies have highlighted the predictive significance of TyG-BMI and TyG-WC in gallstone risk. Research indicates that TyG-BMI (the combination of triglyceride-glucose index and body mass index) and TyG-WC (the combination of triglyceride-glucose index and waist circumference) are significantly associated with gallstone risk, potentially providing more accurate metabolic health information for assessing gallstone risk (25). Thus, the interaction between insulin resistance and RFM may further explain the increased risk of gallstone formation. As an indicator of whole-body fat percentage, RFM not only reflects the metabolic effects of fat accumulation but may also indirectly capture the impact of insulin resistance. In summary, this study, by investigating the association between RFM and gallstones, further elucidates the critical roles of visceral fat accumulation and metabolic dysfunction in the pathogenesis of gallstones.

The findings of this study are consistent with previous research, further confirming the significant correlation between obesity and the incidence of gallstone disease. Prior research has demonstrated that obesity represents an important risk factor for gallstone formation, with BMI and WC widely used as key indicators for assessing obesity levels and predicting gallstone risk (30). However, BMI and WC have limitations in evaluating total body fat and its distribution. As a result, researchers have increasingly focused on novel indicators that can more accurately reflect fat distribution and metabolic health. For example, WWI and BRI are considered to have greater advantages in assessing fat distribution, particularly the degree of visceral fat accumulation (31, 32). These indicators not only better capture whole-body fat percentage and the impact of fat distribution on health but also offer new directions for predicting gallstone risk. Studies have demonstrated that both WWI and BRI are significantly associated with the risk of gallstone formation (17, 33). Additionally, Zhang et al. compared various anthropometric measures and their associations with gallstone risk, finding that BRI and waist-to-height ratio (WtHR) showed the highest predictive value for gallstone risk (34). Thus far, no investigations have delved into the association between RFM and gallstone risk. Through a systematic analysis, this study found that RFM not only more accurately reflects the metabolic consequences of fat accumulation but also outperforms BMI, WC, WWI, and BRI in predicting gallstone risk. This may be because RFM more directly captures the distribution of fat that is closely related to the mechanisms underlying gallstone formation. As a novel assessment tool, RFM provides significant insights for the early screening and intervention of individuals at elevated risk and offers new perspectives for further research on the mechanisms linking obesity to gallstone formation.

The results of this research carry substantial implications for both clinical applications and public health initiatives. RFM, as a practical and accurate assessment tool, can effectively identify high-risk individuals for gallstones, supporting targeted interventions to prevent severe complications such as acute pancreatitis or gallbladder perforation. Additionally, integrating RFM into routine health assessments can enhance the effectiveness of obesity management strategies by focusing on fat distribution rather than solely on weight. From a public health perspective, the use of RFM in large-scale screening programs, particularly in settings with a high prevalence of obesity and metabolic disorders, can improve the efficiency of identifying high-risk populations, thereby facilitating the early prevention and management of gallstones.

This study has several limitations. Firstly, because of its cross-sectional nature, the study cannot ascertain a causal link between RFM and the risk of gallstones. Future longitudinal studies are needed to verify whether elevated RFM directly contributes to the development of gallstones. Second, the identification of gallstones depended on participants’ self-reported information in this study, potentially leading to bias due to recall inaccuracies or incomplete reporting. Additionally, there may be individuals in the general population with occult gallstones who are unaware of their condition and self-report as “no,” which introduces a bias distinct from recall bias. Third, the study sample was based on NHANES data, predominantly reflecting the demographics of the U.S. population. Consequently, additional verification is necessary to ascertain the applicability of the findings to diverse racial or geographical populations. Fourth, due to missing data, participants with incomplete data were excluded from the study, and we were unable to directly compare the characteristics of the excluded and included participants. This could introduce selection bias, especially if the missing data is not completely random (MCAR). Although we used weighted analysis to minimize the impact of such bias, this remains a limitation of the study. Finally, although RFM demonstrated superior predictive ability compared to traditional indices, the further assessment of its clinical applicability and cost-effectiveness across various healthcare environments is still required.

## 5 Conclusion

In summary, this study underscores the notable association between RFM and gallstone risk, demonstrating that RFM outperforms traditional anthropometric measures in gallstone risk prediction. The predictive model constructed by incorporating RFM and other key variables showed good performance, indicating its potential clinical utility. These findings provide strong support for integrating RFM into clinical practice and public health interventions, especially among populations exhibiting a high incidence of obesity and metabolic disorders, offering new perspectives and strategies to reduce the burden of gallstone-related diseases.

## Data Availability

Publicly available datasets were analyzed in this study. This data can be found at: all data for this study were provided by the NHANES website. The dataset is publicly available and can be accessed at the following URL: https://www.cdc.gov/nchs/nhanes/.
