# Supplementary material for: Association between relative fat mass and gallstones: a cross-sectional study based on NHANES 2017–2020
Source: Front Nutr. 2025 Feb 19;12:1554659. doi: 10.3389/fnut.2025.1554659 (PMC11879834; doi:10.3389/fnut.2025.1554659)
Supplement: Supplementary file 1 [file Data_Sheet_1.docx]

**Supplementary Table S1.** Detailed information on categorical covariables.

| Covariable | Category | Explanation |
| --- | --- | --- |
| **Race** | Mexican American | Mexican American |
|  | Other Hispanic | Other Hispanic |
|  | Non-Hispanic White | Non-Hispanic White |
|  | Non-Hispanic Black | Non-Hispanic Black |
|  | Other race | Other race |
| **BMI group** | <25 | Normal or underweight |
|  | 25-30 | Overweight |
|  | ≥30 | Obesity |
| **Education level** | ≤High school | Below 11th grade (including 12th grade without a diploma) |
|  | >High school | High school graduate or above GED equivalent |
| **Physical activity** | Yes | Engage in at least 10 minutes of heart rate-boosting activity weekly. |
|  | No | Avoid 10-minute heart rate-boosting activity weekly. |
| **Smoking status** | Never | Smoked fewer than 100 cigarettes in their lifetime. |
|  | Former | Smoked more than 100 cigarettes but is not currently smoking. |
|  | Current | Smoked more than 100 cigarettes and is currently smoking. |
| **Alcohol** | Yes | Consumed at least 12 drinks of any type of alcoholic beverage in the past year. |
|  | No | Consumed fewer than 12 drinks of alcoholic beverages in the past year. |
| **Diabetes** | Yes | HbA1c level ≥6.5%, or fasting blood glucose ≥126 mg/dL, or self-reported doctor diagnosis, or currently using insulin. |
|  | No | Does not meet the above criteria for diabetes diagnosis. |
| **Hypertension** | Yes | Has been informed by a doctor or other healthcare professional that they have high blood pressure. |
|  | No | Has never been informed by a doctor or other healthcare professional that they have high blood pressure. |

**Supplementary Table S2.** Variance inflation factors for variables.

| Variable | GVIF | Df | GVIF^(1/(2*Df)) |
| --- | --- | --- | --- |
| RFM | 1.593538476 | 1 | 1.26235434 |
| Age | 1.479718202 | 1 | 1.21643668 |
| PIR | 1.325228633 | 1 | 1.15118575 |
| TC | 1.053744403 | 1 | 1.02652053 |
| Race | 1.261628678 | 4 | 1.02947652 |
| BMI group | 1.638803028 | 2 | 1.13144042 |
| Education level | 1.258637406 | 1 | 1.1218901 |
| Physical activity | 1.141981029 | 1 | 1.06863512 |
| Smoking status | 1.229230256 | 2 | 1.05295136 |
| Diabetes | 1.215063925 | 1 | 1.10229938 |
| Alcohol | 1.104327717 | 1 | 1.05086998 |
| Hypertension | 1.327485635 | 1 | 1.15216563 |

**A**


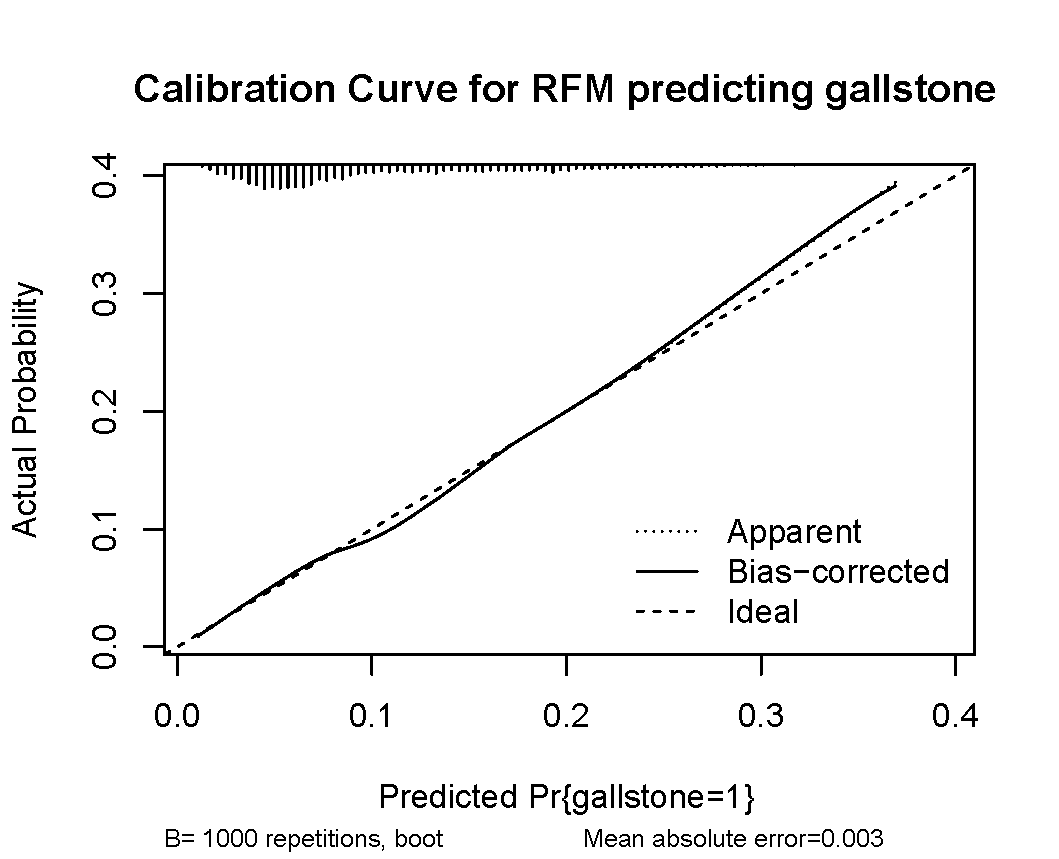


**B**


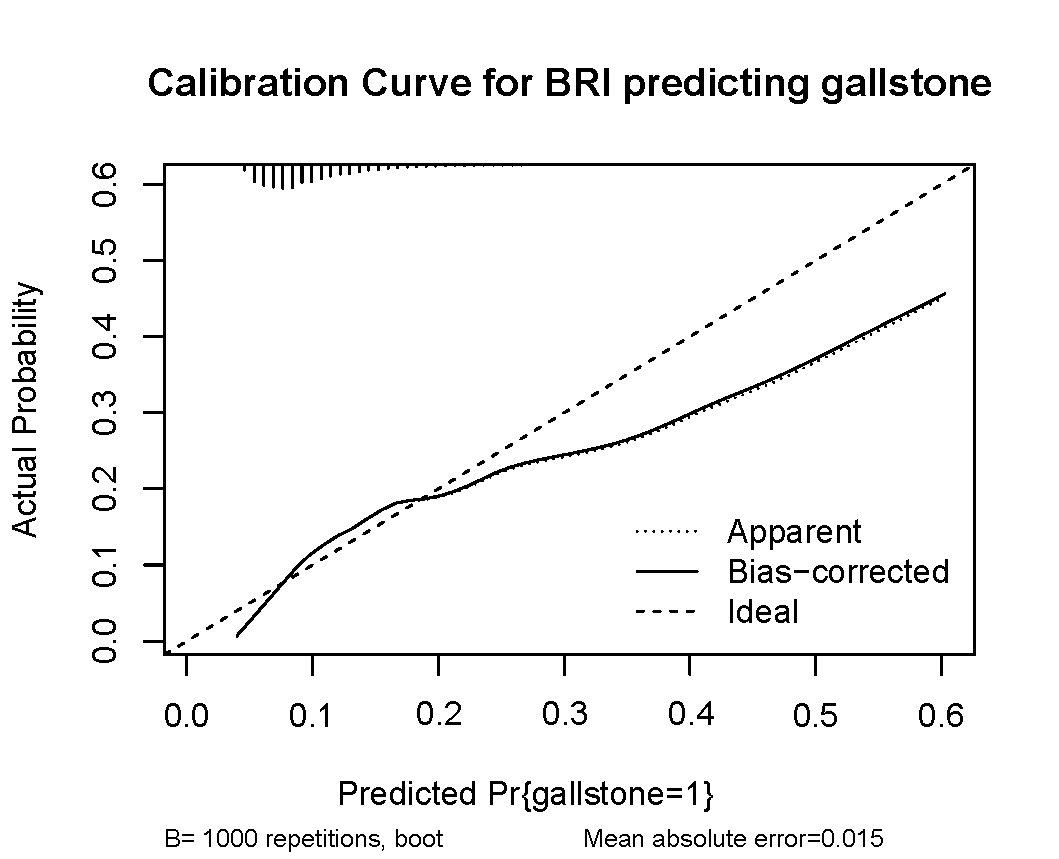


**C**


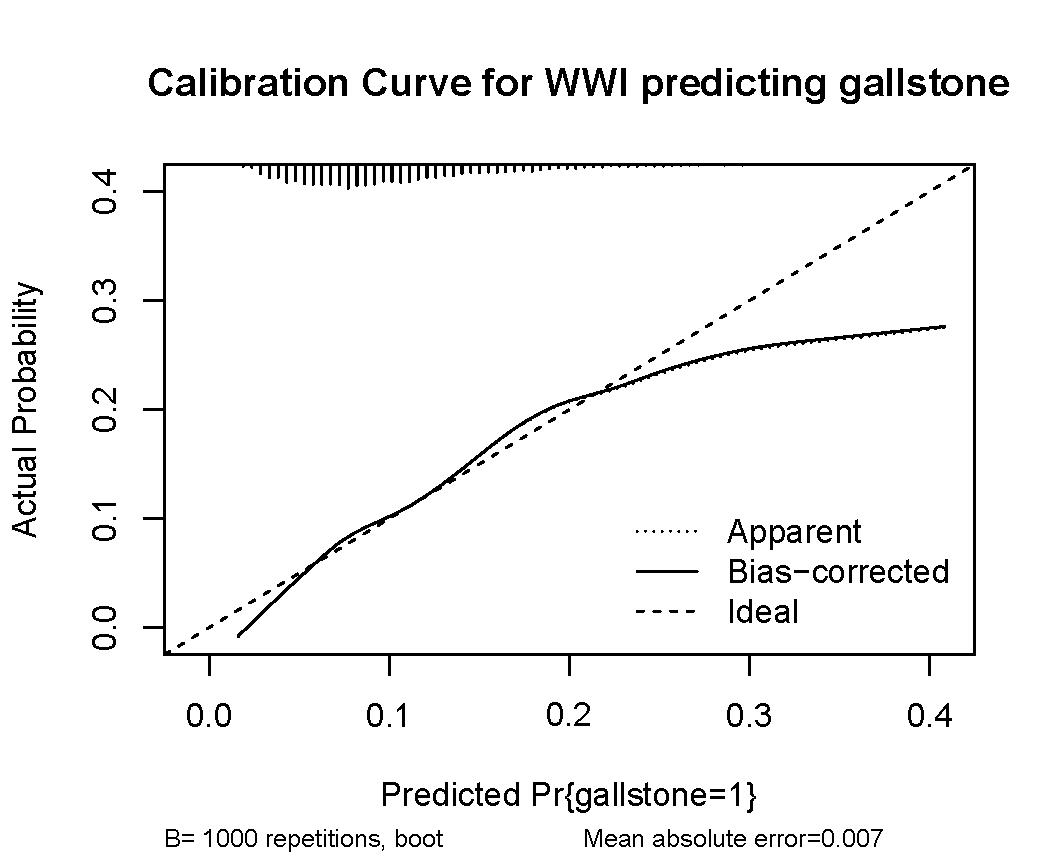


**D**


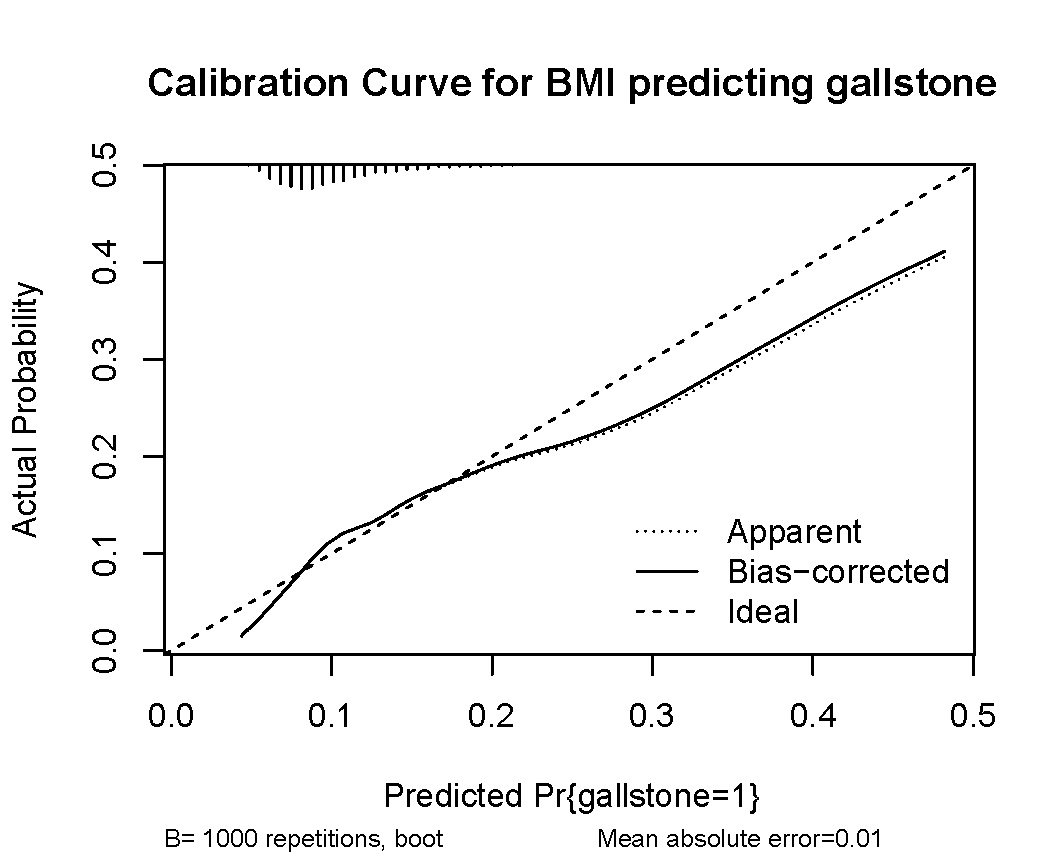


**E**


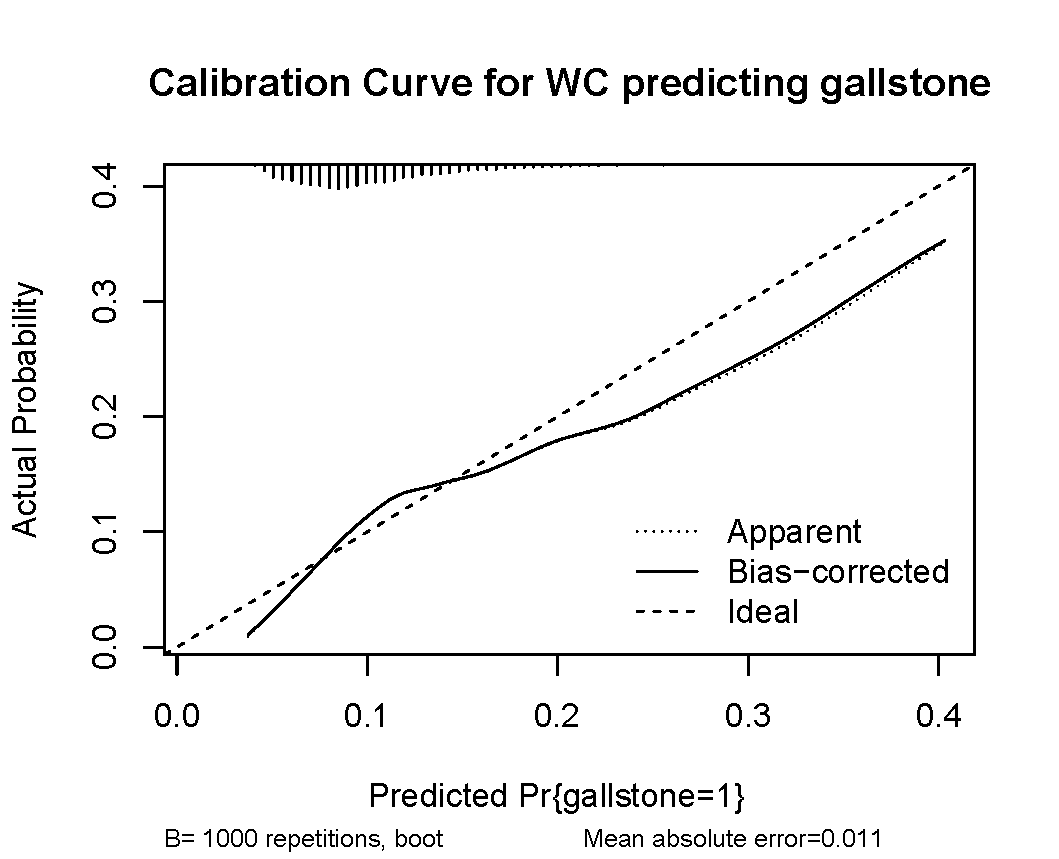


**F**


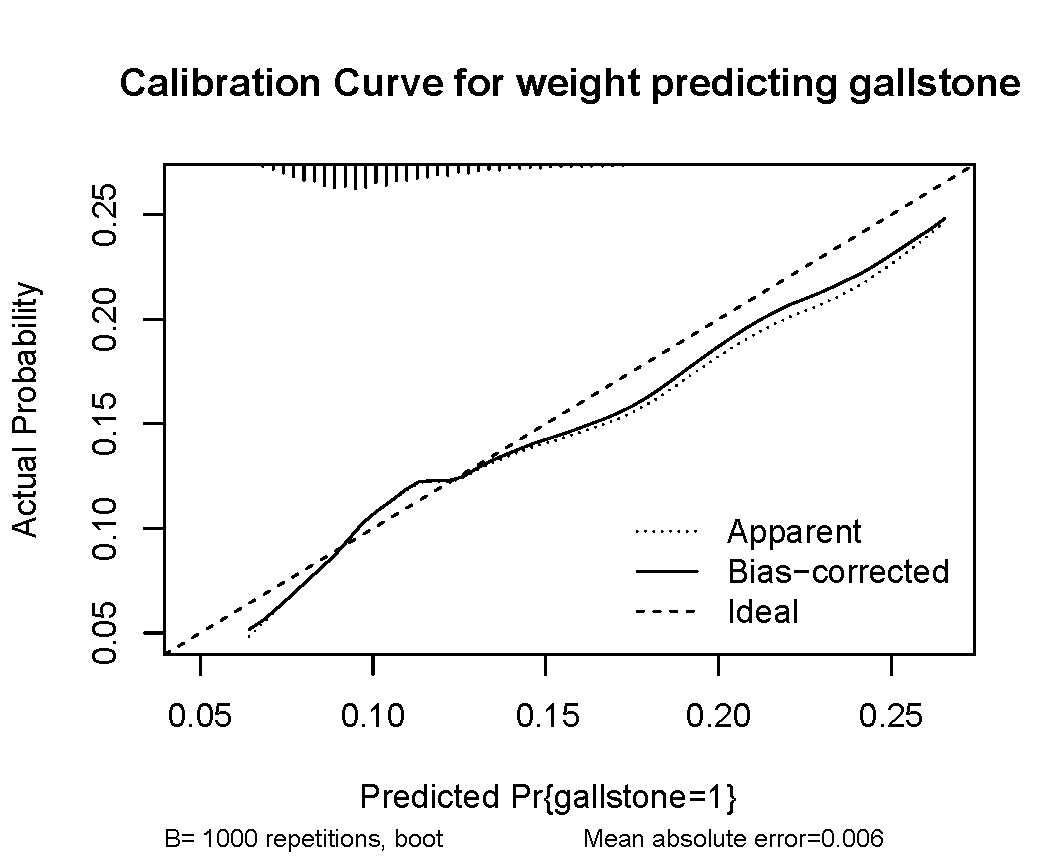


**Supplementary Figure S1.** Calibration curves for predicting gallstone risk using different indices: **(A)** RFM, **(B)** BRI, **(C)** WWI, **(D)** BMI, **(E)** WC, **(F)** Weight.


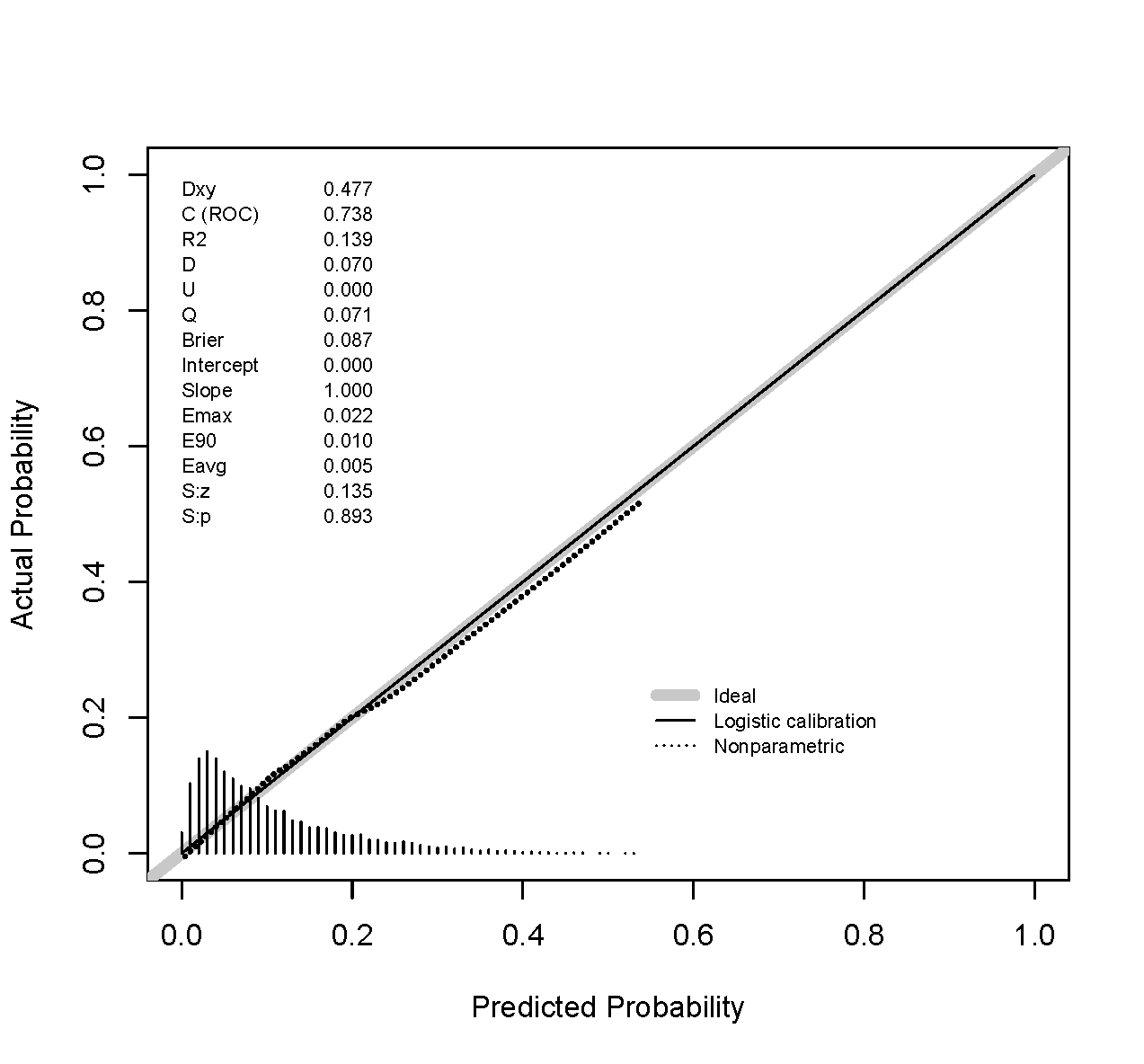


**Supplementary Figure S2.** Calibration Curve for the Gallstone Prediction Model.

**Supplementary Table S3.** Stepwise regression model selection process and its variable coefficients

1. Forward Selection

| Step | Model Formula | AIC |
| --- | --- | --- |
| 1 | Gallstone ~ 1 | 3874.85 |
| 2 | Gallstone ~ RFM | 3577.25 |
| 3 | Gallstone ~ RFM + Age | 3490.66 |
| 4 | Gallstone ~ RFM + Age + Diabetes | 3480.31 |
| 5 | Gallstone ~ RFM + Age + Diabetes + Alcohol | 3479.92 |
| 6 | Gallstone ~ RFM + Age + Diabetes + Alcohol + Weight | 3478.95 |

2. Backward Selection

| Step | Model Formula | AIC |
| --- | --- | --- |
| 1 | Gallstone ~ Gender + Age + BMI + WC + Weight + WWI + BRI + Diabetes + Alcohol + Hypertension + RFM | 3487.22 |
| 2 | Gallstone ~ Gender + Age + BMI + WC + Weight + WWI + Diabetes + Alcohol + Hypertension + RFM | 3485.34 |
| 3 | Gallstone ~ Gender + Age + WC + Weight + WWI + Diabetes + Alcohol + Hypertension + RFM | 3483.71 |
| 4 | Gallstone ~ Age + WC + Weight + WWI + Diabetes + Alcohol + Hypertension + RFM | 3481.92 |
| 5 | Gallstone ~ Age + WC + Weight + WWI + Diabetes + Alcohol + RFM | 3480.64 |
| 6 | Gallstone ~ Age + WC + WWI + Diabetes + Alcohol + RFM | 3479.51 |
| 7 | Gallstone ~ Age + WC + Diabetes + Alcohol + RFM | 3478.89 |

3. Both Directions Selection

| Step | Model Formula | AIC |
| --- | --- | --- |
| 1 | Gallstone ~ Gender + Age + BMI + WC + Weight + WWI + BRI + Diabetes + Alcohol + Hypertension + RFM | 3487.22 |
| 2 | Gallstone ~ Gender + Age + BMI + WC + Weight + WWI + Diabetes + Alcohol + Hypertension + RFM | 3485.34 |
| 3 | Gallstone ~ Gender + Age + WC + Weight + WWI + Diabetes + Alcohol + Hypertension + RFM | 3483.71 |
| 4 | Gallstone ~ Age + WC + Weight + WWI + Diabetes + Alcohol + Hypertension + RFM | 3481.92 |
| 5 | Gallstone ~ Age + WC + Weight + WWI + Diabetes + Alcohol + RFM | 3480.64 |
| 6 | Gallstone ~ Age + WC + WWI + Diabetes + Alcohol + RFM | 3479.51 |
| 7 | Gallstone ~ Age + WC + Diabetes + Alcohol + RFM | 3478.89 |
